# Assessing Short-Term Fertility Intentions and Their Realisation Using the Generations and Gender Survey: Pitfalls and Challenges

**DOI:** 10.1007/s10680-020-09573-x

**Published:** 2020-12-24

**Authors:** Zuzanna Brzozowska, Eva Beaujouan

**Affiliations:** 1 Department of Socioeconomics, Vienna University of Economics and Business, Wien, Austria; 2 Faculty of Social Studies, Masaryk University, Brno, Czechia; 3 University of Vienna, Wittgenstein Centre for Demography and Global Human Capital (IIASA, OeAW, University of Vienna), Wien, Austria

**Keywords:** Fertility intentions, Realisation of fertility intentions, Generations and gender survey, Cross-national survey comparability

## Abstract

The use of fertility intention questions to study individual childbearing behaviour has developed rapidly in recent decades. In Europe, the Generations and Gender Surveys are the main sources of cross-national data on fertility intentions and their realisation. This study investigates how an inconsistent implementation of a question about wanting a child now affects the cross-country comparability of intentions to have a child within the next three years and their realisation. We conduct our analysis separately for women and men at prime and late reproductive ages in Austria, France, Italy and Poland. The results show that the overall share of respondents intending to have a child at some point in their life is similar in all four analysed countries. However, once the time horizon and the degree of certainty of fertility intentions are included, substantial cross-country differences appear, particularly in terms of proceptive behaviour and, consequently, the realisation of fertility intentions. We conclude that the inconsistent questionnaire adaptation makes it very difficult to assess the role of country context in the realisation of childbearing intentions.

## Introduction

In the last forty years, the use of fertility intention questions to study individual childbearing behaviour has developed rapidly. These questions were originally designed to inform forecasting, and the extension of their use to individual studies has led to the emergence of a new strand of population research (Ní Bhrolcháin and Beaujouan 2011). Several theoretical approaches have been developed, which mostly see short-term fertility intentions as direct predecessors of reproductive behaviour (Miller and Pasta 1995). The economic, cultural and institutional constraints that govern both the formation and the realisation of short-term fertility intentions have become one of the core subjects of fertility studies (e.g. Billingsley and Ferrarini 2014; Dommermuth et al. 2015; Hanappi et al. 2017; Hohmann-Marriott 2015; Ní Bhrolcháin and Beaujouan 2019; Philipov et al. 2006; Régnier-Loilier and Vignoli 2011; Thomson 1997).

In Europe, the Generations and Gender Surveys (GGS) are the main sources of cross-national data on fertility intentions and their realisation (Gauthier et al. 2018). They provide data on short- and long-term fertility intentions for 20 countries (including two non-European ones) and on the realisation of short-term fertility intentions in 14 countries. The availability of the GGS data has been a game-changer for fertility researchers. At the same time, the first-completed round of panel data collection suffered from an acknowledged weakness: it allowed the participating countries to introduce changes to the core questionnaire. Despite the efforts made by the GGP team to harmonise the data (Gauthier et al. 2018), the decision heavily compromised data comparability, and questions on fertility intentions were particularly affected. Existing evidence demonstrates that answers to questions on reproductive intentions and preferences are sensitive to question wording and sequencing, as well as to answer options (Beaujouan 2014; Kaufmann et al. 1997; Maddow-Zimet and Kost 2019; Mathews et al. 2012).

This study investigates the cross-country comparability of short-term fertility intentions and their realisation among women and men in Austria, France, Italy and Poland. Specifically, we explore the effect of inconsistent implementation of the preceding relevant question about *wanting a child now* on fertility intentions *within the next three years* and their realisation; we assess how differences in the use and wording of this first question affect the way in which respondents answer the second. In order to properly understand the relationship, we analyse the link between the question wording and proceptive behaviours (i.e. contraceptive non-use) at prime and late reproductive age.

## Data and Analytical Strategy

**Fig. 1:**
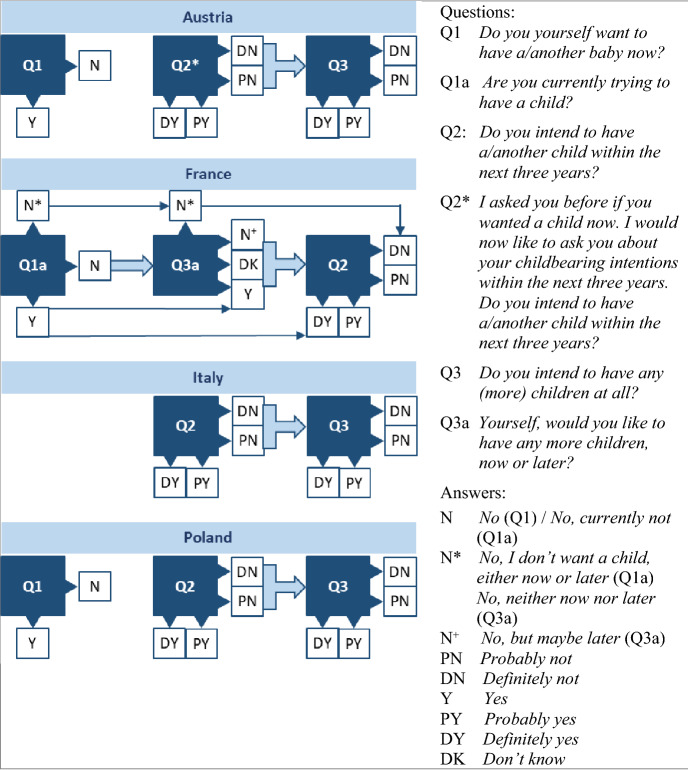
How respondents were asked about short- and long-term fertility intentions in Austria, France, Italy and Poland. *Note:* The order in which the questions are shown corresponds to the order in which they were asked in each country. Wide arrows between answers and questions denote filtering, for example, only respondents who answered PN or DY to Q2 were asked Q3. Thin arrows between answers mean inclusion, for example, all respondents who answered Y to Q1a were automatically classified as answering Y to Q3a and DY to Q2. For France, the answers N* and N^+^ were retrieved from the original national data set. In the harmonised data set, for Q1a N* was recoded as missing; for Q3a, N* was recoded as either DN or missing, whereas N^+^ was distributed among DN, PN, PY, DY and the missing values

The standard GGS core questionnaire included two questions on short-term reproductive decision-making, asked independently of each other: *Do you yourself want to have a/another baby now?* (asked in the survey section “Fecundity”, Q1 in Fig. 1) and *Do you intend to have a/another child within the next three years?* (short-term fertility intention asked in the “Intentions to have children” section, Q2 in Fig. 1). Those who gave a negative answer to Q2 were asked an additional question about their long-term childbearing plans: *Supposing you do not have a/another child during the next three years, do you intend to have any (more) children at all?* (Q3 in Fig. 1). Respondents could answer *yes* or *no* to Q1 and *definitely yes*, *probably yes*, *probably not*, *definitely not* to Q2 and Q3. Our primary interest being short-term fertility intentions, we used the first and second waves of the GGS for Austria, France, Italy and Poland where wording and answer options for Q2 were almost identical to the core questionnaire. The remaining ten countries participating in both GGS waves did not meet this criterion, e.g. there was no time frame in the question about short-term fertility intentions, and the answers to Q2 and Q3 were binary (yes–no); or there were data issues (for instance sample too small, large panel attrition or inaccurate childbearing histories).

Despite the almost identical implementation of Q2 in the four chosen countries, the short-term fertility intentions turned out not directly comparable because of substantial differences in Q1, Q3, the order of questions and/or the filters applied. In Austria and Poland, which fielded the survey when the international questionnaire was already consolidated, the three questions followed the standard questionnaire template, though in Austria an introduction was added to the question on short-term fertility intentions (Q2* in Fig. 1). Italy, which fielded its survey before the standard questionnaire was consolidated, did not ask the question about wanting a child now (Q1). France introduced changes not only in question wording and answer options but also in question order and filters. It replaced the Q1 question with *Are you trying to have a baby now?* (Q1a) and the question about fertility intentions in a more distant future (Q3) with a general question *Yourself, would you like to have any more children, now or later?* (Q3a), which was asked *before* the Q2 question on short-term intentions. Furthermore, Q1a was used as a filter question: those who answered *yes* were automatically classified as answering *yes* to Q3a and *definitely yes* to Q2, whereas those who answered *No, I don’t want a child, either now or later* were automatically classified as answering *no, neither now nor later* to Q3a and *definitely not* to Q2 (Fig. 1). These large deviations from the core questionnaire, only partially captured by the standard GGS harmonisation procedure, led us to retrieve some of the answers to questions about fertility intentions directly from the original French national data set.

In the analysis, we included only panel respondents aged between 18 and 45 and who reported having a partner (cohabiting or not) at wave 1 (see Table 1 in "Appendix" for panel sample characteristics). By selecting only persons in a couple, we ensure maximum possible uniformity of the groups analysed in our study of the realisation of intentions. We first explored how short-term (Q1/Q1a and Q2/Q2*) and overall (including also Q3 and Q3a) childbearing intentions of women and men varied across four age groups: below age 25, 25–29, 30–34 and 35 + . We then closely examined the consequences of different uses and wordings of the “wanting now” question (i.e. the preceding relevant question, Q1/Q1a) on the reported three-year intentions (Q2/Q2*) of respondents of prime reproductive ages (aged below 35) and of late reproductive ages (aged 35 and more). This part of the analysis was conducted only for Austria, France and Poland, as respondents in Italy were not asked whether they want a baby now. We compared the proportion of respondents not using contraceptives among those who reported trying to have a baby (France) and wanting to have a baby now (Austria and Poland). Finally, we determined the realisation rates among three different groups of respondents aged below and above age 35: those with definite short-term fertility intentions (answering *definitely yes* to Q2/Q2*), those with uncertain short-term fertility intentions (answering *probably yes* to Q2/Q2*), and those wanting (Austria and Poland) or trying (France) to have a baby at the time of the first interview (Q1/Q1a). At all stages, the data are weighted with post-stratification weights.

## Results

### Fertility Intentions

**Fig. 2:**
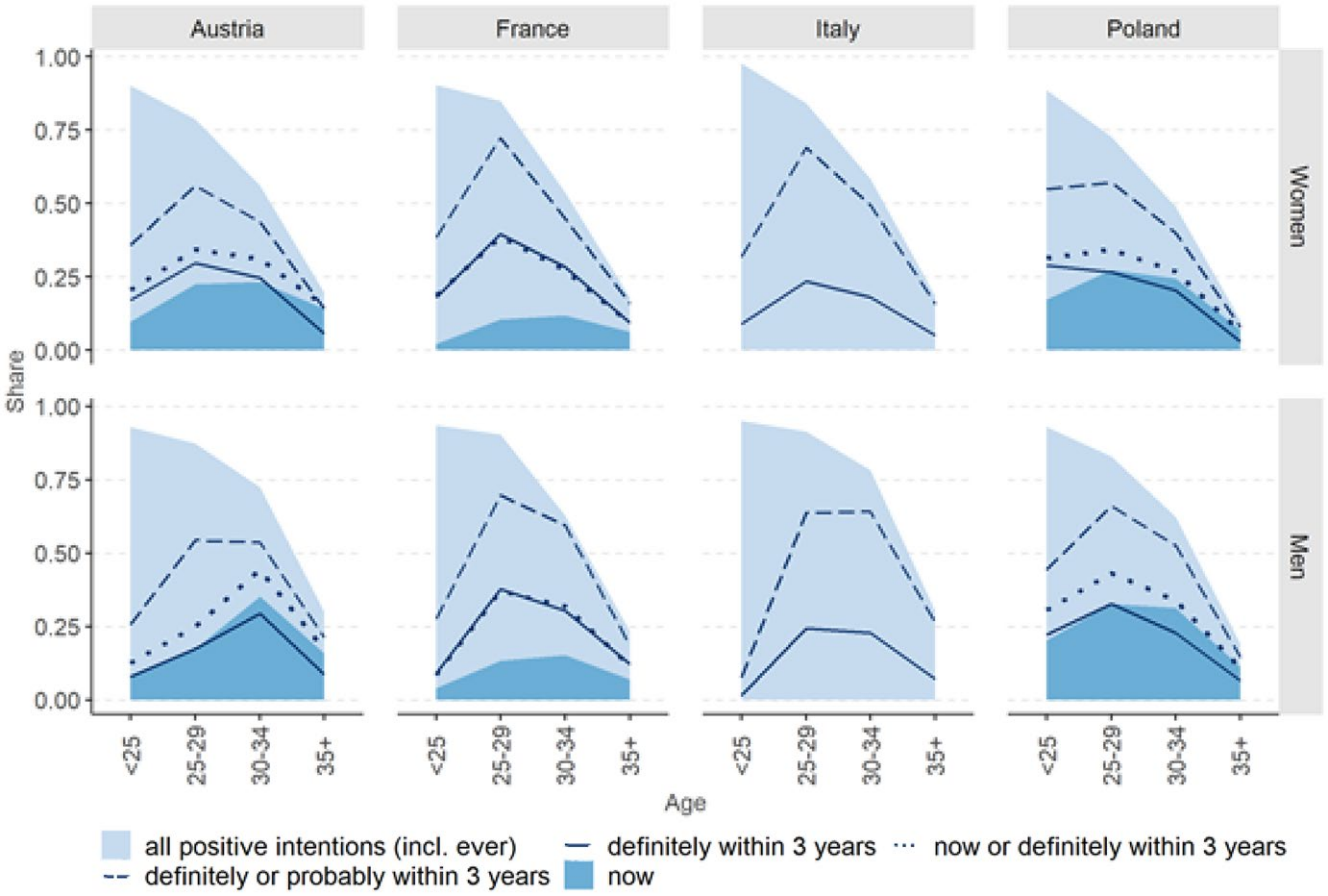
Intentions to have a child by country, sex and age. *Note:* The light-blue area represents the share of respondents who answered either yes to the question “Do you want to have a baby now?” (in France: “Are you trying to have a baby now?”), or probably/definitely yes to the questions “Do you intend to have a/another child within the next three years?” and “Do you intend to have (more) children at all?”. Respondents with missing information on fertility intentions were excluded

Figure 2 shows how the share of women and men probably or definitely intending to have a child either now, within three years or in the more distant future, changes over the life course. In their early 20s, over 90% of respondents in all four countries intend to have a child at some point in their lives (light blue area). The proportion drops sharply in the 30 s as many people already have children; depending on the sex and the country, 10–30% of the respondents aged 35 years and above intend to have a (another) child sometime in their lives.

The short-term fertility intentions curves peak at different levels and ages across countries. The dashed line, which depicts the proportion of respondents who reported definitely or probably intending to have a child within three years, peaks in France and Italy at age 25–29 (more than 60%). In other countries, proportions are lower but somewhat more spread across ages. At the same time, however, Italy has by far the lowest share of respondents definitely intending to have a child in the three-year time horizon (solid line).

**Fig. 3:**
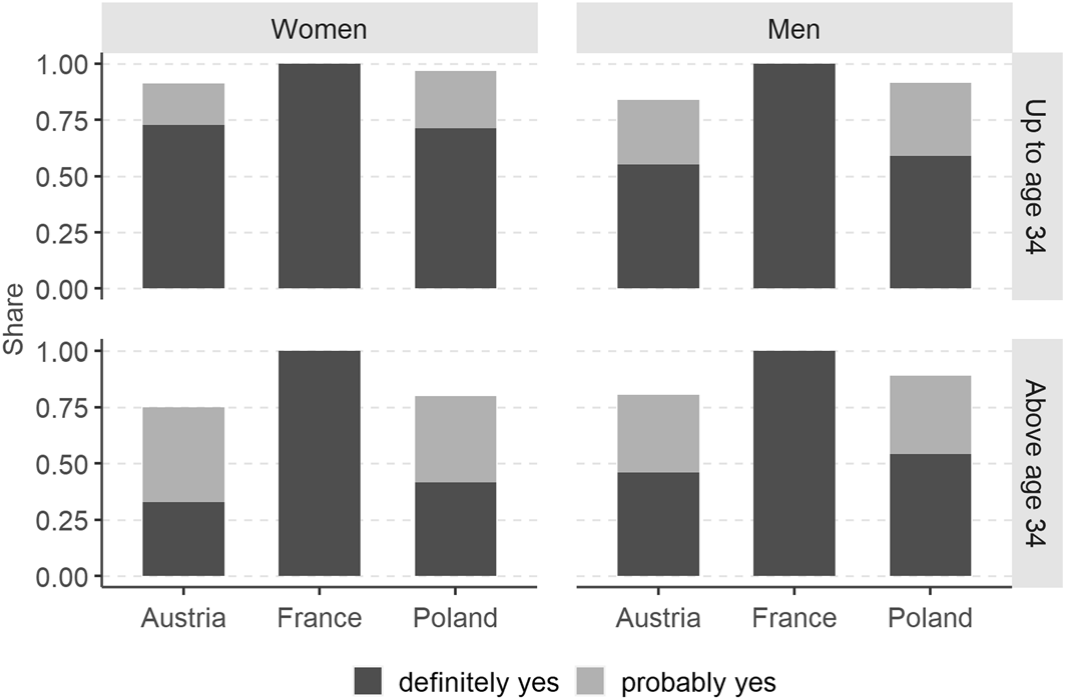
Answers of respondents wanting a child now to the question about childbearing intentions within the next three years, by country, age group and sex. *Note:* In France, all respondents stating they were trying to have a baby now were automatically classified as definitely intending to have a child within the next three years. In Italy, respondents were not asked whether they wanted or were trying to have a baby now

For our purpose, it is instructive to study the intersection between the share “wanting/trying now” (dark blue area) and the share “definitely intending within 3 years” (solid line). In France, those *trying to* have a baby now are all considered as definitely intending to have a child within three years, so that the total of the two groups corresponds to the share definitely intending to have a child. By contrast, in Austria and Poland where the question on *wanting* a baby now was not used as a filter question, the solid line is often inside the dark blue area. In fact, only 50% of men and 75% of women aged below 35 wanting a baby now reported definitely intending to have a child within three years (Fig. 3). In the age category 35+ , the proportions do not change for men but for Austrian and Polish women they drop to 30% and 40%, respectively. Overall, around 15% (age below 35) and 25% (age 35+) of Austrian and Polish respondents who reported wanting a baby now did not report probably or definitely intending to have a child within three years.

### Wanting/Trying Now and Contraception

**Fig. 4:**
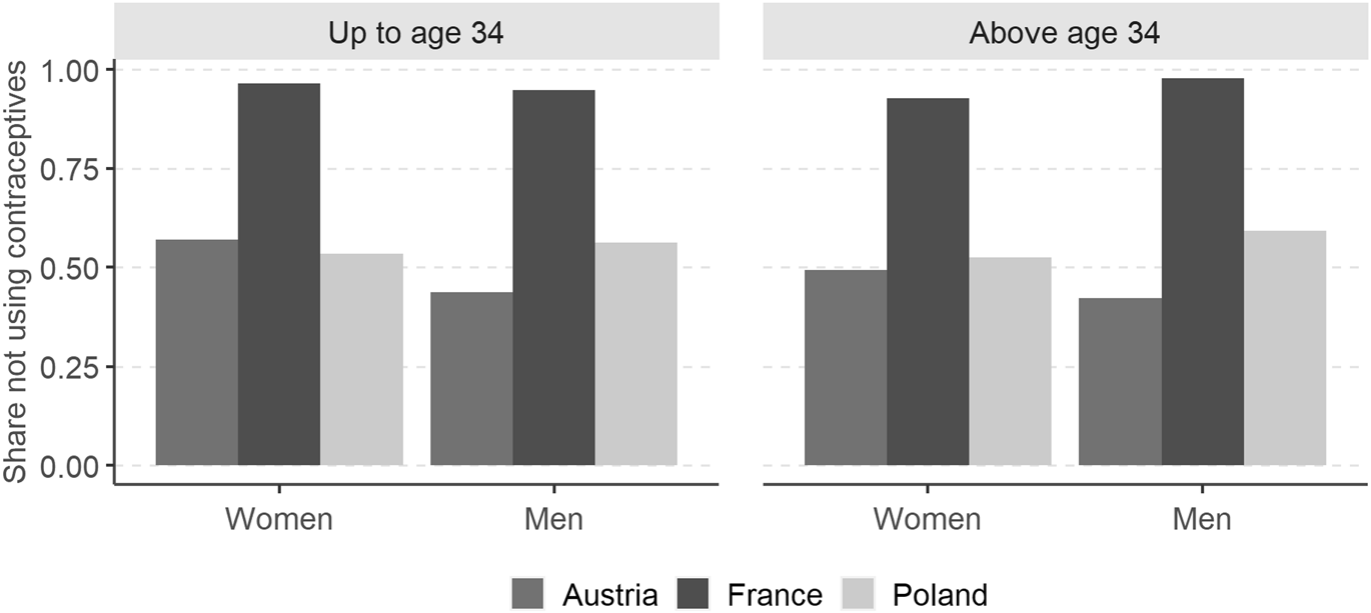
Non-use of contraceptives among women and men intending now (Austria and Poland) and trying now (France), by age group and sex. *Note:* The population in the denominator covers respondents who answered yes to the question “wanting now”/“trying now” in the fecundity section

In addition to the filtering difference, the French Q1a question (trying now) was very different conceptually from the Q1 question asked in the other countries (wanting now). Among women and men who were *trying* to have a baby in France, over 90% were not using contraceptives (Fig. 4). In Austria and Poland, only half of the respondents who reported *wanting* a baby now were not using contraceptives. This clearly illustrates that the question on *wanting now*, though asked at the beginning of the fecundity section, does not correspond to current active attempts to conceive a child and that it is not comparable to the French question on *trying now*.

### Realisation of Fertility Intentions

**Fig. 5:**
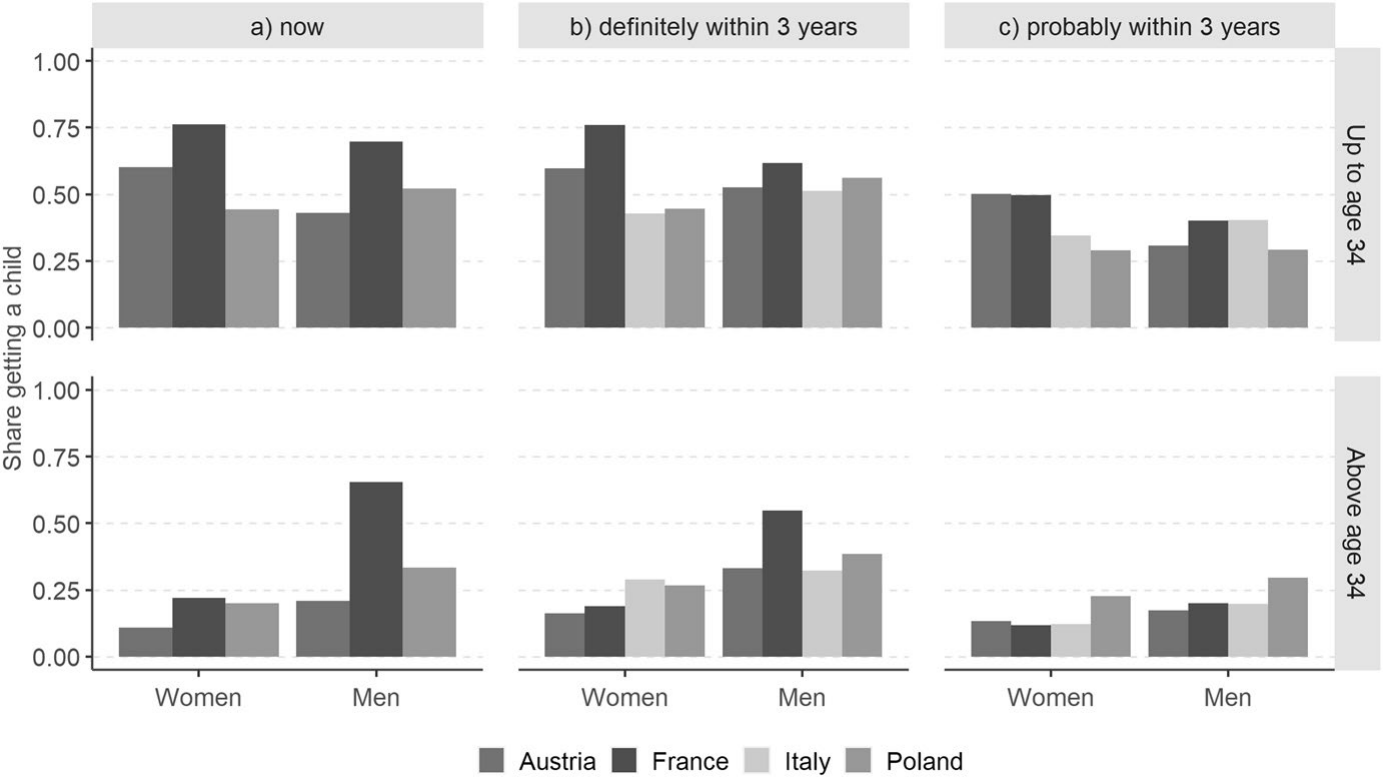
Realisation of short-term fertility intentions by country, age group and sex. *Note:* The population in the denominator covers respondents who answered (**a**) yes to the question “wanting now”/“trying now” of the fecundity section; **b** definitely yes to the question on the intention to have a child within the next three years; **c** probably yes to that same question

Consistent with contraceptive (non-)use, the proportion of respondents who actually have a child among those *trying* to have one is significantly higher than among those *wanting* to have one now, for both men and women (Fig. 5, left-hand panel). The only exception is women aged 35 and more, whose realisation rates are no higher in France than in Austria or Poland (differences in proportions are not statistically significant; significance coefficients are available upon request). Likewise, whereas women below age 35 definitely intending to have a child within three years actually have one substantially more often in France than in other countries, this is not the case for women aged 35 and higher (middle panel of Fig. 5). For men, we see an opposite pattern: French men at prime reproductive age do not differ from their peers in other countries, but those past their 35th birthday have higher realisation rates than in Austria and Italy (and about the same as in Poland). This is probably because the share of men trying to have a baby among those definitely intending to have a child within the next three years increases with age, from 42% in the younger group to 60% in the older one (results available upon request). Among French women, the increase is even sharper, from 30 to 69%, respectively, but as the realisation rates among those trying to have a baby now at age 35+ are no higher than among those wanting a child now in Austria and Poland, the realisation rates among older women with definite short-term fertility intentions in France are not affected.

Ironically, the group with the highest degree of cross-national comparability are respondents known for the weakest link between reproductive intentions and behaviour: those with uncertain short-term fertility intentions (Fig. 5, right-hand panel). Their realisation rates vary relatively little across countries and do not reflect the different implementations of the questions.

## Conclusions

This study aimed to demonstrate how the inconsistent use and wording of a preceding relevant question affects the cross-country comparability of questions about short-term fertility intentions in the GGS. The results show that the overall share of respondents intending to have a child at some point in their life is similar in all four analysed countries. However, once the time horizon and the degree of certainty of fertility intentions are included, substantial cross-country differences appear, particularly in terms of realisation.

Due to the inconsistent adaptation of the survey questionnaire, we are not able to fully assess the role of country context for the realisation of childbearing intentions. For instance, women with definite short-term intentions seem to realise them more frequently in France than in other countries. However, we cannot determine whether this is due to their generally higher fertility or a narrower selection into that question through a more specific preceding question and the fact that it was used as a filter. Therefore, in line with Gauthier et al. (2018), we strongly recommend a coordinated, centralised and highly disciplined data collection that provides truly cross-nationally comparable data.

We also clearly demonstrate that the question “Are you currently trying to have a child?” is much better suited than “Do you want to have a baby now?” for the survey section focusing on *fecundity* and as a preceding relevant question. Particularly, only half of the respondents who gave a positive answer to the “want now” question in Austria and Poland were not using contraceptives. In France, the corresponding figure for those currently trying exceeded 90%. The French question seems to be understood unambiguously by the respondents whereas the Austrian and Polish one, implemented verbatim from the core GGS questionnaire, obviously does not.

In addition, in both Austria and Poland, the overlap between respondents wanting a baby now and definitely intending to have one within three years is surprisingly small, with levels as low as 30% (Austria) and 40% (Poland) among women aged 35+ . The reason for this low-reporting consistency may be found in Miller’s (2011) insights into reproductive decision-making, and the distinction he makes between desires (“to want”) and intentions (“to intend”): “the former simply reflect a wish to achieve a goal through some sort of action, whereas intentions involve a specific decision to pursue an actionable goal, with an associated commitment and, commonly, a plan for implementing the decision” (Miller 2011, p. 78). Clearly, asking respondents about whether they *want* a child *now* tells us much more about respondents’ *current childbearing desire* than about their actual actions undertaken to satisfy that desire.

The low correlation between the answers to these two questions encourages us to assume that the presence or absence of the “want now” question should not affect the answers to the question about short-term fertility intentions. Thus, the fact that the proportion of women who definitely intend to have a child within the next three years is lower in Italy, where the “want now” question was not asked, than in other country can be possibly attributed to other reasons than differences in the questionnaire, for example to the chronic economic and institutional problems that discourage Italians from making definite childbearing plans.

Our study offers two further insights. First, while among respondents at prime reproductive age, the realisation rates are higher for those definitely intending to have a child within three years or wanting one now than for those probably intending to have one, among respondents at late reproductive age this difference disappears. The case numbers at later ages are small, but the pattern is consistent across countries. Possibly, at a later age, higher numbers of women have fecundity problems among those who definitely intend to have a child. Alternatively, women at late reproductive age become more aware of possible age-related sterility and adapt their intentions (Wagner et al. 2019). Consequently, the certainty level becomes less predictive of childbearing behaviour.

Second, among women aged 35+ wanting a child now (Austria and Poland) and trying to have one (France), the share who actually give birth remains far below the estimated biological fecundity at this age (Leridon 2008). This is particularly puzzling in the case of France, where over nine in ten women trying to have a child adopt a proceptive behaviour and where women at younger ages have substantially higher realisation rates than in the other two countries. Furthermore, in Italy, a country with the latest childbearing schedules in the world, the realisation rates among women aged 35+ *definitely* intending to have a child within the next three years are significantly higher than in Austria and France, despite the fact that the figures among Italian women at prime reproductive ages are substantially lower than in these two countries. These two findings suggest that the realisation of fertility intentions at older ages may partly hinge on the prevailing social norms regarding age limits for having children.

## Appendix

See Table A1

**Table A1:** Women and men answering yes, probably yes and definitely yes to the “childbearing intention” and to the “want a child now” questions, and total sample size, unweighted data

	Austria				France				Italy				Poland			
	< 25	25–29	30–34	35 +	< 25	25–29	30–34	35 +	< 25	25–29	30–34	35 +	< 25	25–29	30–34	35 +
Positive childbearing intentions	Women															
Ever (Q3: PY and DY, Q3a: Y and N^+^)	195	234	171	132	177	146	121	92	177	207	243	228	153	217	205	89
Probably within 3 years (Q2 and Q2*: PY)	40	81	57	56	47	54	39	34	35	100	126	134	51	88	82	46
Definitely within 3 years (Q2 and Q2*: DY)	35	89	72	36	37	67	68	52	12	58	83	60	41	84	85	24
Now (Q1 and Q1a: Y)	21	69	72	91	5	19	29	34	–	–	–	–	25	81	92	50
N	235	320	359	768	223	209	292	606	185	242	422	1382	183	376	505	948
Positive childbearing intentions	Men															
Ever (Q3: PY and DY, Q3a: Y and N^+^)	123	172	126	170	59	118	97	84	93	92	191	272	63	152	163	120
Probably within 3 years (Q2 and Q2*: PY)	23	73	42	64	16	38	42	28	6	30	92	170	16	66	76	56
Definitely within 3 years (Q2 and Q2*: DY)	11	37	53	55	7	48	49	46	2	21	62	65	18	56	58	40
Now (Q1 and Q1a: Y)	11	38	62	93	3	19	23	26	–	–	–	–	17	54	69	59
* N*	135	209	203	563	85	150	200	515	102	106	255	1004	75	217	297	705

Sample: women and men in a couple, present at both waves. N gives the sample size by age group
